# Proximity-dependent biotin labelling reveals CP190 as an EcR/Usp molecular partner

**DOI:** 10.1038/s41598-020-61514-0

**Published:** 2020-03-16

**Authors:** Marina Yu. Mazina, Rustam H. Ziganshin, Mikhail D. Magnitov, Anton K. Golovnin, Nadezhda E. Vorobyeva

**Affiliations:** 1 Institute of Gene Biology, Russian Academy of Sciences, Moscow, 119334 Russia; 2 Shemyakin-Ovchinnikov Institute of Bioorganic Chemistry, Russian Academy of Sciences, Moscow, 117997 Russia; 3 Center for Precision Genome Editing and Genetic Technologies for Biomedicine, Moscow, 119334 Russia

**Keywords:** Epigenetics, Transcription

## Abstract

Proximity-dependent biotin labelling revealed undescribed participants of the ecdysone response in *Drosophila*. Two labelling enzymes (BioID2 and APEX2) were fused to EcR or Usp to biotin label the surrounding proteins. The EcR/Usp heterodimer was found to collaborate with nuclear pore subunits, chromatin remodelers, and architectural proteins. Many proteins identified through proximity-dependent labelling with EcR/Usp were described previously as functional components of an ecdysone response, corroborating the potency of this labelling method. A link to ecdysone response was confirmed for some newly discovered regulators by immunoprecipitation of prepupal nuclear extract with anti-EcR antibodies and functional experiments in *Drosophila* S2 cells. A more in-depth study was conducted to clarify the association of EcR/Usp with one of the detected proteins, CP190, a well-described cofactor of *Drosophila* insulators. CP190 was found to co-immunoprecipitate with the EcR subunit of EcR/Usp in a 20E-independent manner. ChIP-Seq experiments revealed only partial overlapping between CP190 and EcR bound sites in the *Drosophila* genome and complete absence of CP190 binding at 20E-dependent enhancers. Analysis of Hi-C data demonstrated an existence of remote interactions between 20E-dependent enhancers and CP190 sites which suggests formation of a protein complex between EcR/Usp and CP190 through the space. Our results support the previous concept that CP190 has a role in stabilization of specific chromatin loops for proper activation of transcription of genes regulated by 20E hormone.

## Introduction

Transcriptional regulation involves the coordination of several hundred proteins in limited space. This feature may have led to associations between many transcriptional regulators and the formation of multisubunit transcriptional complexes, many of which are multifunctional. Protein-protein interactions are essential for the formation of transcriptional complexes^1^. Due to the strength and stability of these interactions, they were successfully detected in chromatography purification experiments of targeted proteins in protein complexes. During transcriptional regulation, many weaker protein-protein interactions take place. Many of these may have functional significance but remain unknown. We assert that contemporary techniques of *in vivo* proximity-dependent labelling can illuminate such interactions.

*In vivo* proximity-dependent labelling technology was developed based on the directed mutagenesis of *Escherichia coli* biotin-ligase BirA^2^. This modified enzyme provides a higher level of promiscuous biotinylation than the original (e.g., an endogenous BirA can specifically biotinylate only the BAP target). The exceptional properties of the modified BirA formed the basis for BioID, a proximity-dependent biotin labelling method^3^. The BioID enzyme is covalently linked to the protein of interest so it can biotinylate its partners in the surrounding protein complex. Next, the modified proteins are precipitated by streptavidin resin and analysed with mass spectrometry (LC-MS/MS) fingerprinting. The BioID technique has rapidly developed in recent years leading to the second generation BioID2, which is a smaller enzyme with enhanced labelling activity^4^. BioID has successfully investigated protein compounds of certain chromatin loci^5,6^. This technique has predominantly been used for the analysis of stable protein agglomerates (e.g., heterochromatin) because BioID labelling requires hours to be affective. This feature makes BioID a poor method for the characterization of protein complexes with a high turnover rate (e.g., promoter-bound transcriptional complexes). Recently, an additional directed mutagenesis procedure of BirA has allowed for the development of the fast TurboID enzyme^7^.

An alternative to the BioID2 technology is a combination of APEX2, a plant ascorbate peroxidase, and a biotin-phenol substrate. In the presence of hydrogen peroxide, the APEX2 enzyme can catalyse the substrate conversion (biotin-phenol) into active and short-lived radicals which can modify the surrounding proteins^3^. Although the method is less common than the BioID technique, APEX2 labelling requires significantly less time to biotinylate surrounding proteins, making it more suited for researching unstable complexes^8,9^. For example, it was recently integrated with the CRISPR/Cas9 system to investigate DNA-protein interactions, as an alternative to ChIP-Seq^10^.

The main research interest of our group is to accurately describe the molecular mechanisms of transcriptional activation in eukaryotes. The ultimate goal is to understand the general mechanism for most genes, including the cooperative function of hundreds of transcriptional factors. As an experimental approach we use *Drosophila melanogaster* developmental genes (e.g., 20-hydroxyecdysone (20E)-dependent), which are a well-regulated physiological system that is convenient to study^11,12^. The main molecular sensor of an ecdysone system is the EcR/Usp heterodimer. It is associated with the regulatory regions of DNA, and bound with co-activators or co-repressors depending on the transcriptional status^13,14^. The change in affinity of EcR/Usp to co-regulators upon transcription was interrogated in our recent work^15^. Using two 20E-dependent genes as an experimental model, we tested the recruitment of 20 different co-regulators to promoters during their activation. We identified that 20E-depedent promoters persist in Pol II pausing prior to transcriptional induction, and were associated with Pol II and other transcriptional regulators. Furthermore, we found that the amount of promoter-bound regulators did not change upon induction of transcription. Our study showed that co-activator recruitment may not cause transcription initiation for 20E-depending genes. We proposed that additional functional input is needed for transcription to commence. This input may be from proteins that were overlooked in previous studies, and may be EcR/Usp co-regulators with weak interactions. We suggested that an association of these weakly bound regulators can in fact be functional and driving activation. Our current study aims to find unknown participants of 20E-dependent promoters using proximity-dependent protein labelling.

## Materials and methods

### Treatment of *Drosophila* S2 cells

*Drosophila* Schneider cell line 2 (S2) cells were maintained at 25 °C in ecdysone-free Schneider’s insect medium (Sigma) containing 10% FBS (HyClone). The treatment of S2 cells with 20-hydroxyecdysone (20E) (H5142, Sigma-Aldrich) was performed at a final concentration of 0.3 μM, as was described previously^15,16^.

BioID2 as well as GS 25 nM linker were amplified from the MCS-13X Linker-BioID2-HA plasmid, which was a gift from Kyle Roux (Addgene plasmid #80899; http://n2t.net/addgene:80899; RRID:Addgene_80899)^4^. APEX2 were taken from mito-V5-APEX2 plasmid which was a gift from Alice Ting (Addgene plasmid #72480; http://n2t.net/addgene:72480; RRID:Addgene_72480)^9^. Both BioID2 and APEX2 enzymes were fused to EcR (isoform A) or Usp through a 25 nm GS linker.

For the stable expression S2 cells were transfected with the corresponding expression plasmids (all fusions were placed under the Act5C promoter and marked with 3xFLAG epitope) and the pCoBlast plasmid, taken at a 20 to 1 molar ratio using the Effectene Transfection Reagent (Qiagen). Polyclonal cell lines bearing stably integrated expression constructs were selected and maintained subsequently in Schneider medium supplemented with Blasticidin (Sigma-Aldrich) at a final concentration of 20 μg/mL.

Immunoprecipitation^11^ and RNA interference-mediated knockdowns of CP190, Chro, Mor, Brm, Mi-2, NELF A, Spt5 and Nup358 in *Drosophila* S2 cells^15^ were performed as previously described. Efficiency of RNA knockdown was evaluated by qPCR and provided at Fig. S1.

### Proximity-dependent biotin labelling procedures

S2 cells expressing BioID2-fused proteins were treated with 20E or solvent (DMSO) for 14 hours prior to protein extraction. Biotin were added at a 50 nM final concentration into cultural medium for all treatment period to enhance labelling. The nuclear fraction was obtained after a 15-min incubation of cells in a low-salt buffer (20 mM Hepes KOH, pH 7.9, 5 mM MgCl_2_, 10 mM KCl, protease inhibitor cocktail (Roche)), followed by centrifugation at 700 g for 7 min, as described previously (Mazina *et al*., 2018). The nuclear fraction was incubated in the Lysis buffer (20 mM Hepes KOH, pH 7.9, 5 mM MgCl_2_, 100 mM KCl, 0.1% NP40, DNase I (ThermoScientific), protease inhibitor cocktail (Roche)), for 10 min at 4 °C and subjected to two rounds of ultrasonication, 10 sec each. Nuclear extracts were centrifuged for 20 min at 14,000 g and used for precipitation with Streptavidin–Agarose (S1638, Sigma-Aldrich). After 2 hours of incubation at 4 °C on a rotating platform, IPs were washed three times with the Lysis buffer and one with 1xPBS. Precipitates were eluted with the PAGE loading buffer (50 mM Tris-HCl, pH 6.8, 2% SDS, 10% glycerol, 100 mM dithiothreitol), and analysed by western blots or subjected to trypsin treatment and LC-MS/MS analysis.

S2 cells expressing APEX2-fused proteins were treated with 20E or solvent (DMSO) for 1 hour and with Biotin-Phenol (SML2135, Sigma-Aldrich) for 30 minutes prior to protein extraction. 5 minutes prior to nuclear extraction S2 cells were treated with 1 mM H_2_O_2_. To stop the APEX2 labelling, 10 mM NaN_3_, 5 mM Trolox and 10 mM Sodium ascorbate were supplemented into low-salt buffer, which was used to extract nuclear fraction. Remaining steps of biotin-labelled proteins extraction were exactly the same as in experiment with BioID2-fused proteins.

Full lists of precipitated proteins, identified by LC-MS/MS fingerprinting, are provided in Supplementary dataset files. The most prominent EcR/Usp protein partners were selected for the Tables 1 and 2 using the following criteria: (1) more than 10 (than 20 for APEX2) detected peptides at least in one condition (EcR-sham, Usp-sham, EcR-20E, Usp-20E); (2) more than 2.5 enrichment over maximal negative control (sham or 20E-treated)at least for one condition; (3) only proteins which were previously identified as a nuclear were selected. Quantitative comparison of precipitated proteins after DMSO- of 20E-treatment is also provided as Supplementary dataset files (only proteins which changed their amount in precipitations upon 20E treatment are listed).

**Table 1:** Proximity-dependent biotin ligation via BioID2 reveals both described and novel molecular partners of EcR and Usp.

Gene	Protein_ID	USP-BioID 20E sham	USP-BioID 20E 14 h	EcR-BioID 20E sham	EcR-BioID 20E 14 h	BioID 20E sham	BioID 20E 14 h
*Nuclear pore subunits*							
*Nup*3*58*	A0A0B4K7J2	49	38	40	20	1	1
*Nup50*	Q7K0D8	18	19	13	6	1	0
*Nup*2*14*	Q9W1X4	14	16	15	3	2	1
***Nup88***	Q9GYU8	6	6	12	4	2	0
*Chromatin remodeling complexes*							
***Mor***	A0A0B4JDA0	12	13	9	4	0	0
*Chromatin architectural proteins*							
*Chro*	Q8T9D1	23	21	10	9	1	3
***CP190***	Q24478	15	12	9	4	0	0
***Pzg***	Q9VP57	18	18	10	5	0	0
*Others*							
***Hcf***	Q9V4C8	38	40	26	16	1	2
***Uba1***	Q8T0L3	29	23	6	2	0	0
***scra***	Q9V4P1	24	23	16	16	2	3
*Dsh*	P51140	17	15	13	8	0	0
*Lam*	P08928	16	17	14	5	0	0
*NELF A*	Q86NP2	15	10	6	2	0	0
*Dp1*	Q7KN75	14	13	24	13	0	0
*CtBP*	O46036	11	6	5	2	0	0
*cg10077*	Q8MZI3	6	3	27	11	0	4
*bel*	Q9VHP0	6	2	22	4	4	6
*CG14712*	Q9VGL0	6	3	15	5	5	4
*Rm62*	P19109	5	2	11	4	0	0

A list of proteins precipitated by streptavidin-agarose from *Drosophila* S2 cells expressing EcR and Usp fused to BioID2 enzyme as estimated using LC-MS/MS fingerprinting. Cells were treated with 20E for 14 hours (or sham-treated for the same period) prior to protein extraction. Peptides count is provided for each protein in all tested conditions. % coverage as well as calculated score are provided in Supplementary datasets with the lists of less prominent precipitated proteins. Names of proteins precipitated using both BioID2 and APEX2-based techniques are marked with Bold.

**Table 2:** Proximity-dependent biotin ligation via APEX2 reveals both described and novel molecular partners of EcR and Usp.

Gene	Protein_ID	USP-APEX sham	Usp-APEX 20E 1 h	EcR-APEX sham	EcR-APEX 20E 1 h	APEX sham	APEX 20E 1 h
*Nuclear pore subunits*							
*Nup205*	Q8IQV9	25	7	21	18	6	8
***Nup88***	Q9GYU8	11	5	21	22	3	6
*Chromatin remodeling complexes*							
*Osa*	A0A0B4KHB1	29	17	16	15	9	10
*Mi-2*	E1JI46	23	9	17	15	5	9
*Brm*	M9PFM5	21	15	8	3	5	10
*kis*	B7Z002	33	15	4	6	6	7
***Mor***	Q9VF03	31	18	12	12	12	12
*Chromatin architectural proteins*							
***CP190***	Q24478	27	15	10	6	10	5
***Pzg***	Q9VP57	23	15	11	14	8	10
*Others*							
*E(bx)*	E1JHV6	37	12	6	2	8	10
*RPII215*	D0Z769	27	14	23	19	1	12
***Hcf***	Q9V4C8	29	20	29	32	8	10
***Uba1***	Q8T0L3	24	15	40	38	9	16
*Ars2*	A0A0B4KEI5	24	17	22	26	13	12
*Sin*3*a*	A0A0B4K765	24	12	15	12	7	8
*Nipped-B*	E1JGX3	24	6	8	10	1	5
*l(*3*)72Ab*	Q9VUV9	23	8	10	5	3	6
*Prp8*	A1Z8U0	23	5	6	3	4	4
*Spt6*	N0D8I3	23	2	6	8	1	2
*RnrL*	P48591	21	13	38	41	15	15
*sle*	Q8INM3	20	8	14	17	1	4
*SMC2*	Q7KK96	20	9	9	14	5	5
***Scra***	Q9V4P1	18	16	37	32	12	8
*smid*	P91638	15	6	21	17	4	7
*CG4119*	Q9V3Y5	15	5	17	22	4	7
*hyd*	A0A0B4LGZ6	14	7	24	22	4	6
*Rpd3*	Q94517	13	11	17	20	6	8
*RanBPM*	A0A0B4K851	12	3	17	20	4	7
*HDAC4*	Q9VYF3	11	3	39	35	6	12
*Uba2*	Q7KJV6	9	2	15	21	2	2
*REG*	Q9V3P3	9	7	12	13	6	6
*CG10489*	A9UND8	7	1	21	21	2	3
*Mcm5*	Q9VGW6	7	3	21	21	4	7

A list of proteins precipitated by streptavidin-agarose from *Drosophila* S2 cells expressing EcR and Usp fused to APEX2 enzyme as estimated using LC-MS/MS fingerprinting. Cells were treated with 20E for 1 hour (or sham-treated for the same period) prior to protein extraction. Peptides count is provided for each protein in all tested conditions. % coverage as well as calculated score are provided in Supplementary datasets with the lists of less prominent precipitated proteins. Names of proteins precipitated using both BioID2 and APEX2-based techniques are marked with Bold.

### ChIP-Seq

The chromatin immunoprecipitation (ChIP) was performed exactly as previously described^17^. ChIP-Seq libraries were obtained using the NEBNext DNA library preparation kit (New England Biolabs). Only the library fragments of 250–500 bp were subjected to NGS sequencing. New generation sequencing was performed by Evrogen with the Illumina NovaSeq6000 sequencer. For each of the ChIP‐Seq libraries, approximately 8–14 million unique paired mappable reads were obtained. The FastQ format paired‐end reads that were obtained were mapped to the Drosophila genome assembly dm6 using Bowtie2^18^ and filtered (with minimum MAPQ quality score = 10).

The EcR and Usp binding sites in *Drosophila* S2 cells were defined by MACS2 with the following parameters: -gsize ‘120000000’ -keep-dup ‘1’-qvalue ‘0.01’ -mfold ‘5’ ‘30’–bw ‘375’ 2 > &1 > macs2_stderr^19^. Corresponding input DNA was used as a control for peak calling. Protein binding profiles were generated using BamCompare which is a part of deepTools2 (protein binding level were normalized to genome content – calculated as RPGC: number of reads per bin/(total number of mapped reads * fragment length/effective genome size)^20^. Protein binding levels were calculated as an enrichment of ChIP-Seq relative to Input DNA. Pile-up profiles were calculated as median level of protein binding for distributions of EcR-BioID2, EcR-APEX2, BioID2 and APEX2 on EcR-bound sites and distributions of Usp-BioID2, Usp-APEX2, BioID2 and APEX2 on Usp-bound sites. For distribution of CP190 binding levels in 20E- or DMSO- treated Drosophila S2 cells (1 hour treatment) on EcR-bound sites and 20E-inducible enhancers and distribution of CP190 on genes in Drosophila S2 cells expressing FLAG-EcR after 1 hour treatment with 20E or in sham-treated cells pile-up profiles were calculated as mean level.

The Galaxy-P platform was used for analysis of ChIP-Seq data^21^. All obtained ChIP-Seq data were deposited into the Gene Expression Omnibus - GSE139316.

### Analysis of Hi-C data

Hi-C data obtained previously for *Drosophila* S2 cells were used for the analysis^22^.

Averaged contact domain between CP190 peaks and enhancers/transcription start sites (TSSs) was calculated as follows: we used the closest CP190 peak at a distance >25 kb from the TSS or enhancer to create a list of potential interactions. The average contact domain was calculated using coolpup.py v0.8.6 with rescale and local options on 5 kb contact matrices^23^. The rescale size was set to 99 pixels and 10 randomly shifted control regions were used to normalize the signal for local background.

Averaged spatial interactions between CP190 peaks and enhancers/TSSs were calculated as follows: we generated all potential pairwise cis contacts between the TSS and enhancers with CP190 peaks of length <1 Mb. These pairs were used for the average loop calculation. The average loop was calculated using coolpup.py v0.8.6 on 5 kb contact matrices with pad size of ±100 kb around the loop pixel and 10 randomly shifted control regions to normalize the signal for local background. The mean value of the central 3×3 pixels represents the average loop strength (or the enrichment of contacts in the loop) and is highlighted in the top left corner of the pile-up.

### Antibodies

Anti-FLAG antibodies were purchased from Sigma-Aldrich (F1804). Antibodies against various nuclear proteins were described previously: anti-EcR^11^; anti-Chro^24^; anti-Rpb3^15^, anti-Mor^15^, anti-Brm^15^, anti-Mi2^15^, anti-Spt5^15^.

For the ChIP-Seq analysis of CP190 we used previously well-described antibodies against CP190 raised in rats^25^. For the immunoprecipitations and immunostaining we used our rabbit antibodies against CP190 which were generated using the same epitope as previously to raise antibodies in rats: amino acid residues 606–1096^25^ (Supplementary figure to illustrate the specificity of rabbit polyclonal antibodies against CP190 using RNAi treatment of S2 cells is provided in Fig. S2). Antibodies against NELF A using epitope corresponding to 943–1248 amino acid residues were generated in this study (The specificity of antibodies against NELF A was characterized in Fig. S2). The epitopes for antibody production were expressed as 6 × His-tagged fusion proteins in Escherichia coli, affinity-purified on Ni Sepharose 6 Fast Flow (GE Healthcare), according to the manufacturer’s protocol, and injected into rabbits, following the standard immunization procedure. Antibodies were affinity-purified using the same epitopes as were used for immunization. Antibodies production was performed according to procedures outlined in the NIH (USA) Guide for the Care and Use of Laboratory Animals. The protocol used was approved by the Committee on Bioethics of the Institute of Gene Biology of the Russian Academy of Sciences. All procedures were performed under conditions designed to minimize suffering.

Anti-lamin ADL67.10 antibodies were provided by Developmental Studies Hybridoma Bank (deposited to the DSHB by Fisher, P. A.).

Antibodies against Nup358 were a generous gift of D. Kopytova (Institute of Gene Biology RAS).

## Results

### EcR and Usp fused to APEX2 and BioID2 enzymes bind genomic sites of EcR and Usp

To reveal previously undescribed molecular partners of the EcR/Usp heterodimer, we used proximity-dependent biotin ligation (based on the BioID2 and APEX2 enzymes). The main advantage of this technique is the ability to elucidate short-term and weak protein-protein interactions, which may still be functionally significant^3^. This approach can supplement previous results from the conventional chromatographic purification of the EcR protein complex^26^.

**Figure 1:**
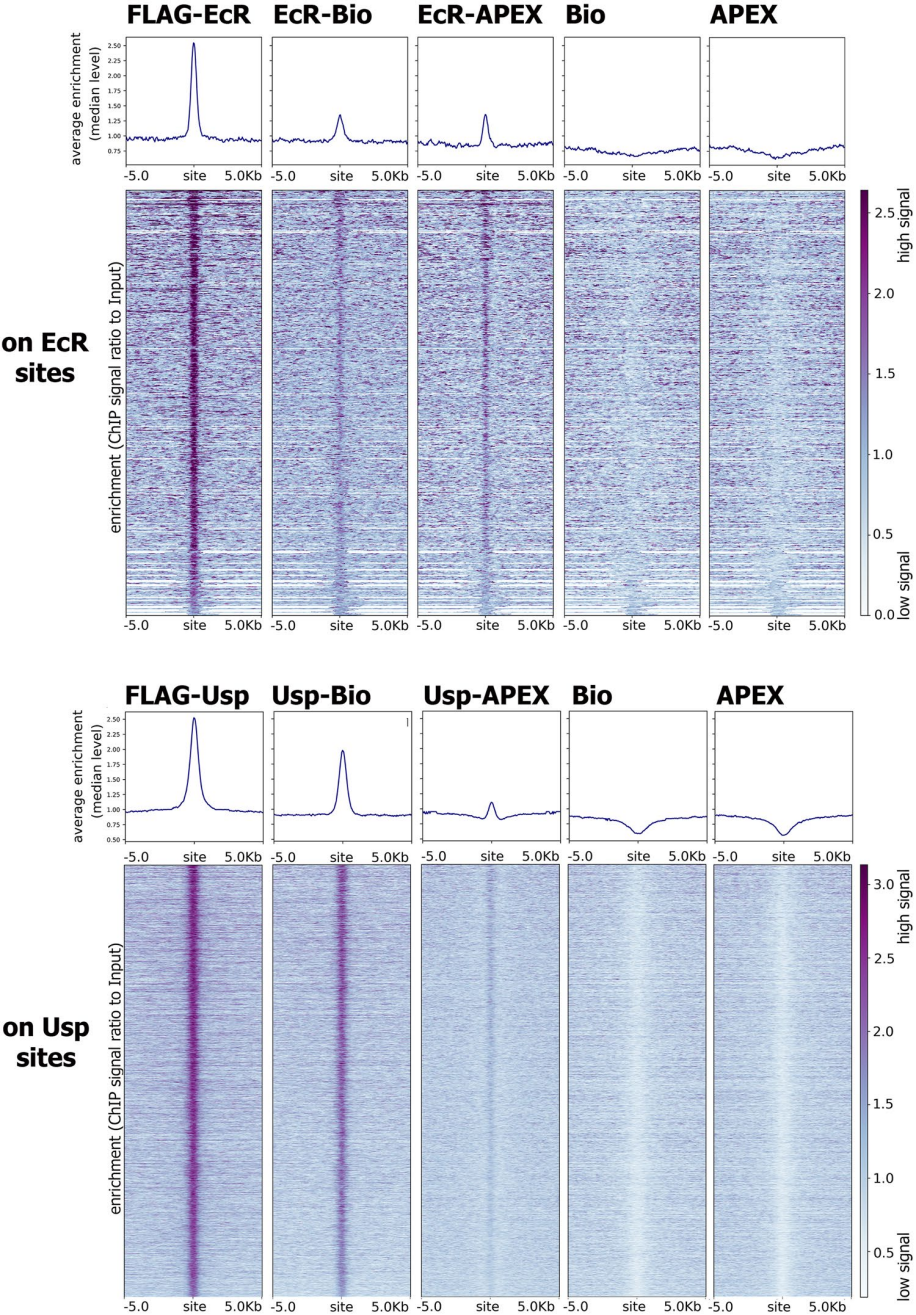
EcR and Usp fused to APEX2 and BioID2 enzymes bind genomic sites of EcR and Usp. (**A**) Distribution of EcR-BioID2 (EcR-Bio), EcR-APEX2 (EcR-APEX), BioID2 (Bio) and APEX2 (APEX) on EcR-bound sites (which were defined using control *Drosophila* S2 cell line expressing EcR in relation to the corresponding input DNA) in *Drosophila* S2 cells after 1-hour treatment with 20E. (**B**) Distribution of Usp-BioID2 (Usp-Bio), Usp-APEX2 (Usp-APEX), BioID2 (Bio) and APEX2 (APEX) on Usp-bound sites (which were defined using control *Drosophila* S2 cell line expressing Usp in relation to the corresponding input DNA) in *Drosophila* S2 cells after 1-hour treatment with 20E. Protein binding level was estimated by ChIP-Seq using anti-FLAG antibodies. Heatmaps represent enrichment of ChIP-Seq signal over Input DNA (presented as the ratio of the number of reads at a given point). Pile-up profiles were calculated as a median of binding levels of corresponding proteins in a given distance out from the binding site.

BioID2 and APEX2 enzymes were marked with a 3xFLAG epitope and fused to EcR and Usp through a flexible 25 nm GS linker^4^. All fused proteins were expressed using the Act5C promoter in *Drosophila* S2 cells. To ensure functionality of the generated proteins, we verified their ability to bind EcR and Usp sites in the *Drosophila* genome (Fig. 1). A list of EcR- and Usp-bound sites in S2 cells after 1 hour treatment with 20E was obtained using ChIP-Seq data for the 3xFLAG-EcR and 3xFLAG-Usp proteins, which were expressed as expected. Details of the ChIP-seq analysis and site selection are provided in the Methods section. Figure 1 depicts the enrichment of EcR fused with BioID2 and APEX2 enzymes on EcR sites and Usp fusions on Usp sites. Our results demonstrate that EcR and Usp fusions can be recruited to genomic sites endogenous for the EcR/Usp proteins. Conversely, unfused BioID2 and APEX2 enzymes do not bind to EcR/Usp sites. The generated EcR-BioID2, Usp-BioID2, EcR-APEX2, and Usp-APEX2 are fully functional proteins capable of binding EcR and Usp genomic targets, making them suitable for proximity ligation assays.

### Proximity-dependent biotin ligation reveals both described and novel molecular partners of EcR and Usp

Our initial goal was not only to describe the molecular partners of EcR and Usp, but to uncover proteins associated with EcR/Usp after 20E treatment. For this assay, we performed experiments of BioID2- and APEX2-based biotin-labelling on 20E- or DMSO- (solvent) treated *Drosophila* S2 cells^12^. It is well established that BioID2- and APEX2-based proximity ligation assays have significantly different kinetics. BioID2 needs at least 6–12 hours for biotin ligation, while APEX2 labelling completes in minutes^3^. These labelling characteristics force us to design different protein purification experiments for the BioID2- and APEX2-fused proteins. S2 cells expressing BioID2-fused proteins were treated with 20E or solvent for 14 hours prior to protein extraction, while cells expressing APEX2 fusions were incubated with 20E for 1 hour. Procedures of nuclear protein extraction and streptavidin-based purification were identical for all proximity-dependent biotin ligation experiments (see Methods). Streptavidin-precipitated proteins were trypsinized and identified with LC-MS/MS. Lists of precipitated proteins are provided in Tables 1 and 2 (and in Supplementary dataset files). The difference in properties of the labelled enzymes as well as the protocol timeline possibly resulted in the different experimental outputs. We assert that proteins modified with the BioID2 enzyme represent partners stably connected with EcR/Usp while APEX2 fusions labelled proteins with a higher turn-over rate.

We identified a significant number of proteins from both assays: Mor, Hcf, CP190, Uba1, Pzg, Scra, and Nup88. Our experiments identified the proximate location of the EcR/Usp heterodimer to chromatin remodelling proteins (Mor, Osa, Brm, Mi-2, and Kismet), nuclear pore proteins (Nup358, Nup214, Nup88, Nup50, and Nup205), and chromatin architectural proteins (CP190, Chro and Pzg). These findings support previous results on the functional involvement of these proteins in the ecdysone response^15,27–29^.

A quantitative comparison of DMSO- and 20E-treated samples was carried out by estimating the relative peak area for the top 3 peptides of each protein (see Supplementary dataset files). Unfortunately, we failed to detect much difference between protein samples precipitated from 20E treated and untreated cells (listed in Tables 1 and 2). This result indicates that the EcR/Usp heterodimer persists in a stable protein complex state. The response to the 20E signal is functional, but does not alter the protein composition of the neighbourhood.

**Figure 2:**
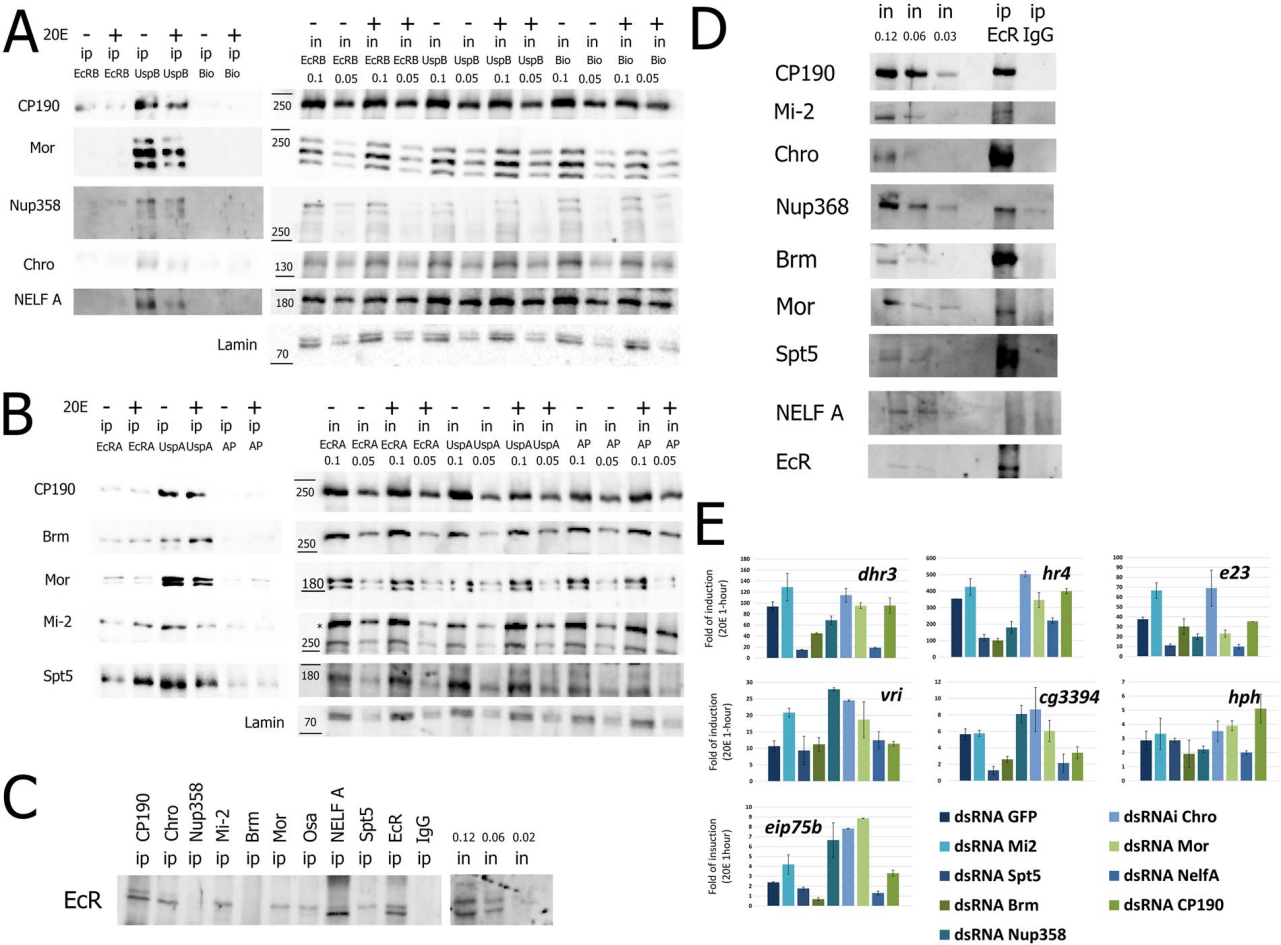
Several candidate partners of EcR/Usp revealed by proximity-dependent labeling were proved to interact with EcR by co-immunoprecipitation and participate in the ecdysone response. (**A**) Streptavidin-based precipitation (ip) from nuclear lysates of Drosophila S2 cells stably expressing EcR-BioID2 (EcR-B), Usp-BioID2 (Usp-B) and BioID2 (Bio) after 14 hours of treatment with 20E (+). Sham-treated (DMSO) cells of the corresponding cell lines were taken as a control (−). Unprecipitated nuclear fraction (in) was also loaded. Western blots were stained with antibodies against CP190, Mor, Nup358, Chro and NELF A. All IP samples were loaded on a single western blot and developed with the same exposure as input samples. Numbers above the inputs represent a portion of a loaded fraction (in respect to amount used for the immunoprecipitations). (**A**) Streptavidin-based precipitation (in) from nuclear lysates of Drosophila S2 cells stably expressing EcR-APEX2 (EcR-A), Usp-APEX2 (Usp-A) and APEX2 (Ap) after 1-hour treatment with 20E (+). Sham-treated (DMSO) cells of the corresponding cell lines were taken as a control (−). To biotin-label the proteins full APEX2-based labeling protocol was performed as described in Methods. Western blots were stained with antibodies against CP190, Mor, Brm, Mi-2, Spt5. All IP samples were loaded on a single western blot and developed with the same exposure as input samples. Numbers above the inputs represent a portion of a loaded fraction (in respect to amount used for the immunoprecipitations). (**C**,**D**) Immunoprecipitations from nuclear protein extracts of *Drosophila* prepupa (0–12 h after puparium formation). Immunoprecipitations were performed with antibodies against CP190, Chro, Mor, Brm, Mi-2, NELF A, Spt5, and Nup358 (**C**) or EcR (**D**) (a serum of non-immunized rabbits (ip IgG) were used as a negative control). Western blots were stained with the corresponding antibodies indicated on the left of the figures. All input and IP samples were loaded on a single western blot. Numbers above the inputs represent a portion of a loaded fraction (in respect to amount used for the immunoprecipitations). (**E**) Transcriptional induction levels of 20E-dependent genes in 20E- treated *Drosophila* S2 cells relative to DMSO-treated (20E treatment was performed for 1 hour). The impact of candidate partners of EcR/Usp on 20E response was analyzed using RNA interference-mediated knockdown (CP190, Chro, Mor, Brm, Mi-2, NELF A, Spt5, Nup358) with dsRNA corresponding to GFP transcript used as a negative control. Transcriptional levels were assessed by qRT-PCR. The Y-axis units represent fold of transcription induction. Data are mean values from three independent experiments, error bars represent standard deviations.

To confirm LC-MS/MS-based identification, we performed western blot analyses on streptavidin-precipitated samples with antibodies against some proteins identified in Tables 1 and 2 (Fig. 2A,B). Staining the streptavidin-precipitated proteins confirmed the results obtained with LC-MS/MS. All tested proteins were found to be enriched in precipitates from cells expressing an EcR/Usp-fusion but not from BioID2 and APEX2 controls. We detected less precipitated proteins in EcR-based experiments, which we explain by a lower expression rate of EcR-fusions in compare to Usp-fusion. For a few proteins we observed a change of the signal upon S2 cells treatment with 20E, but the level of this change does not exceed two times. Thus, we detected only small quantitative but not qualitative changes in composition of a precipitated complex upon 20E treatment.

### EcR and its potential partner proteins co-immunoprecipitate from prepupal nuclear extract

Proximity-dependent biotin labelling of a target protein with BioID2 or APEX2 fusions implies a close location (~1 nm) between bait and target, and does not necessarily indicate a protein-protein interaction but does indicate a possibility of one. To support our findings from biotin-labeling experiments, we tested interactions between EcR and some of the co-immunoprecipitated proteins. As the ecdysone cascade is most active during *Drosophila* puparium formation, we performed our experiments using protein nuclear extract of the prepupa (0–12 h after puparium formation). Antibodies against some potential EcR partners (e.g. CP190, Chro, Mi-2, Mor, NELF A, and Spt5) have successfully co-precipitated EcR from the nuclear extract (Fig. 2C). In a reciprocal experiment antibodies against EcR also co-precipitated these proteins (Fig. 2D). Of the proteins we analyzed, only Brm, Nup358 and Nelf A have demonstrated only one-way co-immunoprecipitation and failed to demonstrate it vice versa. Our results corroborate the potency of our strategy to find EcR partners using labelling methods. Indeed, a large proportion of interactions detected via biotin-labeling was reproduced by co-immunoprecipitation of extracts from a living organism (i.e. not only in cultural cells).

### RNA interference-mediated knockdowns of EcR partner proteins misregulate the 20E response in *Drosophila* S2 cells

To evaluate the functional input of discovered EcR partners we used *Drosophila* S2 cells. The response of S2 cells to 20E has been described previously in detail^12,15^. *Drosophila* S2 cells were treated with 0.3 μM of 20E for an hour to induce transcription of ecdysone-dependent genes. Knockdown of proteins of interest was performed using RNA interference with a dsRNA to GFP as a negative control (Fig. 2E). All analyzed EcR partners (CP190, Chro, Nup358, Mi-2, Mor, Brm, NELFA and Spt5) were found to influence the transcriptional response of S2 cells to 20E treatment. Some of them were shown to constrain transcriptional output (Mi-2 anf Chrom) while others were important for 20E-dependent genes to produce full level of inducibility (Brm, NELF A and Spt5). The rest (Nup358, CP190 and Mor) were found to play roles of co-repressors or co-activators depending on a particular inducible gene.

These results demonstrate functional involvement of analyzed proteins in the 20E response and support their validity as EcR molecular partners. Altogether, our experiments have uncovered several novel physical and functional interactions of EcR (with CP190, Nup358, Chro, Mor, NELF A, and Spt5) and additionally confirmed the previously described interaction with Mi-2^27^.

### CP190 and EcR co-immunoprecipitate from *Drosophila* S2 cells

To complement our approach of screening for the EcR/Usp molecular partners, we investigated in detail the relationship between the heterodimer and one of the candidate genes, the chromatin architectural protein CP190. CP190 was strongly enriched in streptavidin-based precipitations from S2 cells expressing Usp-BioID2 and Usp-APEX2 fusions. EcR-based labelling generated weaker results and was only increased for the BioID2 technique as compared to the control (see Table 1 and Fig. 2A). However, CP190 and EcR were found to co-immunoprecipitate in nuclear extract from *Drosophila* prepupa (Fig. 2C,D).

Another factor for choosing this protein was the previously demonstrated functional involvement of CP190 in the ecdysone response^29^. Our newly obtained data on the approximate location of CP190 to EcR and Usp in the nucleus supports the previous functional data and advances our understanding of the role of CP190 in ecdysone signalling.

**Figure 3:**
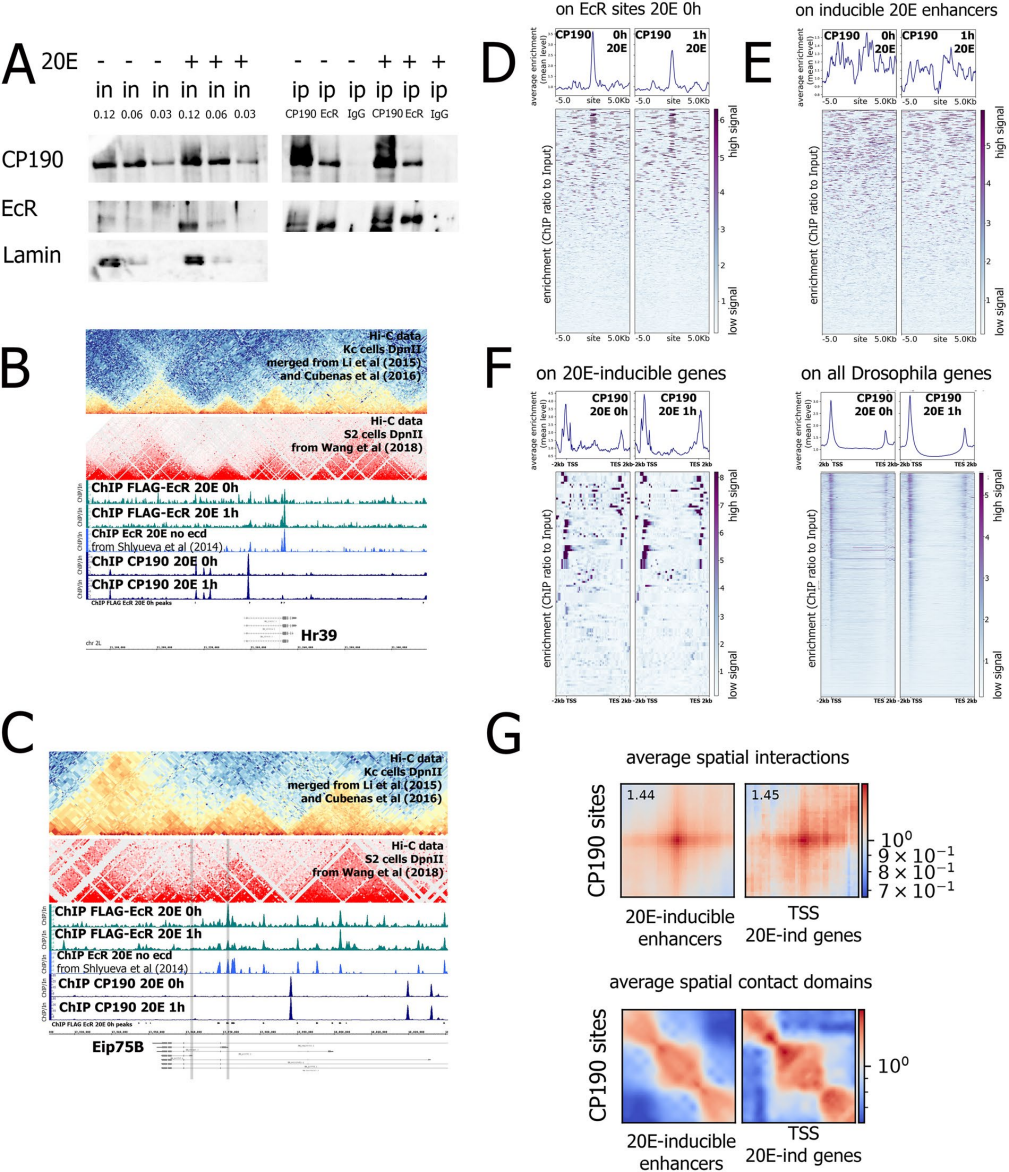
CP190 co-immunoprecipitates with EcR and is present at 20E-dependent loci. (**A**) Immunoprecipitations from nuclear protein extracts of 20E- or DMSO-treated *Drosophila* S2 cells (1 hour treatment) were performed with specific antibodies against EcR and CP190 or a serum of non-immunized rabbits (ip IgG). Western blots were stained with corresponding antibodies indicated on the left of the figures. All IP samples were loaded on a single western blot and developed with the same exposure as input samples (only input samples for the EcR are provided with a greater exposure). Numbers above the inputs represent a portion of a loaded fraction (in respect to amount used for the immunoprecipitations). (**B**,**C**) Binding profiles of EcR and CP190 on 20E-inducible gene loci *hr39* (**B**) and *eip75b* (**C**). Protein binding levels were estimated by ChIP-Seq. ChIP-Seq experiments obtained in the current study were performed using a 20E- or DMSO-treated *Drosophila* S2 cell line expressing 3xFLAG-EcR (1 hour treatment). For the ChIP-Seqs of EcR and CP190 anti-FLAG or specific anti-CP190 antibodies (ChIP FLAG-EcR or ChIP CP190, respectively) were used. For the comparison we also provide previously published data of ChIP-Seq experiments in *Drosophila* S2 cells with the specific anti-EcR generated by Shlyueva and colleagues (without 20E treatment - ChIP EcR no ecd)^30^. We also provide sub-kb resolution Hi-C data obtained for the *Drosophila* S2 cells by Wang and colleagues^22^. Protein binding levels in ChIP-Seqs are provided as an enrichment of ChIP-Seq signal over input DNA. Grey areas at the *eip75b* locus mark 20E-dependent promoters of this gene. (**D**,**E**) Average distribution of CP190 binding levels in 20E- or DMSO- treated *Drosophila* S2 cells (1 hour treatment) on EcR-bound sites (**D**) or 20E-inducible enhancers estimated previously by Shlyueva and colleagues by STARR-Seq^30^ (**E**). A list of EcR-bound sites was estimated with MACS2 peak caller using 3xFLAG-EcR ChIPseq data with the corresponding input obtained from DMSO-treated S2 cells (N = 591). Protein binding levels were estimated by ChIP-Seq using anti-CP190 antibodies. Heatmaps represent enrichment of ChIP-Seq signal over input DNA (presented as ratio of the number of reads at a given point). Pile-up profiles were calculated as a mean of binding levels of corresponding proteins in a given distance out from the binding site. (**F**) Average distribution of CP190 on genes in *Drosophila* S2 cells expressing FLAG-EcR after 1 hour treatment with 20E (1 h 20E) or in sham-treated cells (control S2). Average profiles were defined for genes whose transcription is induced in *Drosophila* S2 cells upon 20E treatment – S2 20E induced (defined in^16^). CP190 binding levels were estimated as an enrichment of CP190 ChIP-Seq signal over input DNA. Pile-up profiles were calculated as a mean of CP190 binding level. Average profiles were generated using metagene mode (introns were ignored). (**G**) Analysis of 3D interactions between the CP190 site and regulatory sites of 20E-dependent genes (enhancers and TSSs) using Hi-C data with a sub-kb resolution obtained previously from *Drosophila* S2 cells^22^. A list of CP190-bound sites was estimated with MACS2 peak caller using our ChIP-Seq data with the corresponding input obtained from DMSO-treated S2 cells. For this analysis enhancers induced by 20E (defined by STARR-Seq previously) and transcriptional start sites of genes induced upon 1 hour treatment of *Drosophila* S2 cells with 20E were used^16,30^. We estimated the average spatial interactions and average spatial contact domains between CP190 peaks and promoters/enhancers of 20E-dependent genes with a Coolpup.py program. Detailed information is provided in Methods.

In co-immunoprecipitation experiments we decided to investigate the impact of 20E hormone in stabilization of the interaction between CP190 and EcR. We performed a co-immunoprecipitation experiment on nuclear protein extracts from *Drosophila* S2 cells with specific anti-CP190 and anti-EcR antibodies (Fig. 3A). We successfully co-immunoprecipitated EcR protein by anti-CP190 and vice versa from the *Drosophila* S2 cells treated with 20E for an hour and from the untreated cells. We conclude from these results that CP190 forms multiprotein complex with EcR both in 20E untreated and treated conditions.

### CP190 is present at only a portion of EcR-bound sites in the genome, but CP190 sites interact with regulatory regions of 20E-dependent genes

As CP190 was observed to form a multiprotein complex with the EcR/Usp heterodimer, we investigated whether CP190 occupies the same sites as EcR/Usp. We performed CP190 ChIP-Seq analysis using *Drosophila* S2 cell lines expressing 3xFLAG-EcR in a 20E- or solvent-treated state (Fig. 3B,C). A list of EcR-bound sites was estimated with MACS2 peak caller using 3xFLAG-EcR ChIP-Seq data with the corresponding input obtained from 20E-untreated S2 cells (N = 591)^19^. The estimated list of sites was compared to the previously published ChIP-Seq results obtained in S2 cells using specific EcR antibodies^30^. MACS2 peak calling on previously published EcR ChIP-Seq data (without 20E treatment) revealed 140 EcR bound sites of which 92 overlapped with the sites bound with 3xFLAG-EcR. We found a good correlation between the two datasets in strong peaks located in 20E-dependent genes with a lesser correlation in weaker peaks (Fig. 3B,C). We associate this effect with a slightly different binding profile generated by overexpressed 3xFLAG-EcR protein in relation to endogenous EcR.

We found presence of CP190 at only a portion of EcR-bound sites in either condition (20E- or DMSO- treated) (Fig. 3D). Next, we evaluated CP190 binding levels at the 20E-dependent (induced) enhancers estimated previously for *Drosophila* S2 cells using STARR-Seq analysis (Fig. 3E)^30^. We found a complete absence of CP190 binding at the STARR-Seq peaks demonstrating that observed CP190-bound EcR sites are not the 20E-dependent enhancers. This result was in agreement with a recent study in *Drosophila* Kc cells, where CP190 binding was found at EcR sites which are not enriched with enhancer markers H3K9me1, H3K27Ac, and CBP^31^.

Finding very modest correlation between EcR and CP190 in genome binding and having convincing experimental data on the existence of these proteins in a multiprotein complex, we decided to look deeper into organization of 20E-dependent genomic loci in regard to EcR and CP190 binding (Fig. 3B,C). We observed CP190 binding on transcriptional start and end sites of some 20E-dependent genes (Fig. 3B). This type of binding is well correlated with a pile-up profile of CP190 binding on 20E-dependent genes induced in S2 *Drosophila* cells. A mean level was calculated to obtain an average profile of CP190 across the genes (Fig. 3F). Distribution of CP190 binding across the 20E-dependent genes was very close to the profile CP190 has at an average gene of Drosophila genome. Still for a great portion of 20E-dependent loci we did not observe CP190 binding at the transcriptional start and end sites (Fig. 3F,C).

It was previously suggested that CP190 plays a role in 3D organization of 20E-dependent loci to promote proper enhancer-promoter interactions^29^. The impact of CP190 on 3D structure of 20E-dependent Eip75B loci was demonstrated by 3C analysis^29^. We decided to additionally test this hypothesis and performed analysis of 3D interactions between the CP190 site and regulatory sites of 20E-dependent genes (enhancers and promoters) using Hi-C data with a sub-kb resolution obtained previously from *Drosophila* Kc and S2 cells^22,32,33^. Inspecting Hi-C matrices across the 20E-dependent loci we observed that promoters and enhancers of 20E-dependent genes often form domains enriched in interactions with CP190 sites nearby (Fig. 3C). We decided to analyze 3D relations of CP190 with regulatory regions of 20E-dependent genes at an average level.

For this analysis we used enhancers induced by 20E that were revealed previously by STARR-Seq and transcriptional start sites of genes induced upon 1 hour treatment of *Drosophila* S2 cells with 20E^16,30^. We estimated average spatial interactions and average spatial contact domains between CP190 peaks and promoters/enhancers of 20E-dependent genes with a Coolpup.py program (Fig. 3G)^34^. It was observed that regulatory sites of 20E-dependent genes indeed interact with close CP190-bound sites forming domains enriched in internal interactions (Fig. 3E,G). Thus, depending on the particular 20E-dependent gene, CP190 can participate in the formation of regulatory hub of 20E-dependent gene loci out of (1) promoter, (2) common site bound both with EcR and CP190 or (3) out of the distant CP190-bound site (by the looping mechanism).

### Mutation in *cp190* delays pupariation and misregulates transcription of 20E-dependent genes

**Figure 4:**
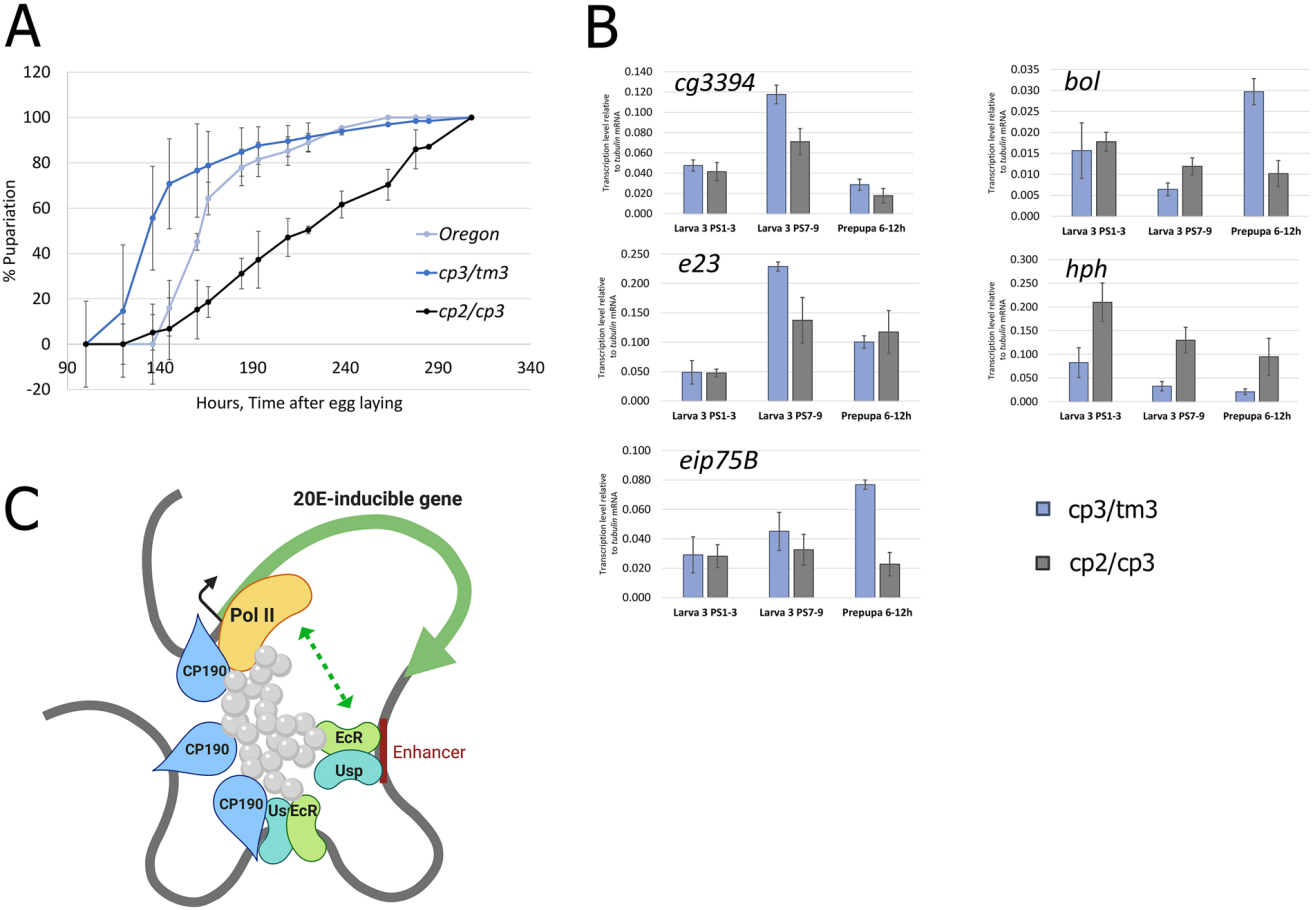
Mutation in *cp190* delays pupariation and misregulates transcription of 20E dependent genes. (**A**) The larval-to-pupal transition was analyzed in loss-of-functional mutants of *cp190* (*cp190*^*2*^*/cp190*^*3*^) and in control flies (Oregon or *cp190*^*3*^*/tm3*) flies. Pupariation was calculated by observing the percentage of pupariated animals after egg laying (provided in %). (**B**) Transcription levels of the 20E-dependent genes in Larva PS 1–3 (eating), Larva PS 7–9 (pupariated) and in Prepupa 6–12 h after puparium formation of *cp190* LOF mutants (*cp190*^*2*^*/cp190*^*3*^) or control flies (*cp190*^*3*^*/tm3*). Transcriptional levels were assessed by qRT-PCR. The Y-axis units represent transcription level relative to tubulin mRNA. Data are mean values from three independent experiments, error bars represent standard deviations. (**C**) Proposed model of an average 20E-dependent gene describing a role of CP190 in transcriptional regulation of 20E-dependent loci (image created with BioRender.com). Depending on the particular gene, CP190 can participate in the formation of regulatory hub out of (1) promoter, (2) common site bound both with EcR and CP190 or (3) distant CP190-bound site (by the looping mechanism).

CP190 was previously shown to be functionally involved in 20E-dependent regulation, mediating transcription of some 20E-dependent genes in *Drosophila* Kc cells (Wood *et al*., 2011). In order to additionally demonstrate the functional input of CP190 in a 20E-dependent response, we analysed the 20E-mediated larva-prepupa transition in loss-of-functional (LOF) mutants of *cp190*. Pupal lethality has been established for many *cp190* alleles, indicating the role of CP190 into pupation^35,36^. We detailed its role further by measuring the timing of puparium formation in *cp190*^2^*/cp190*^3^ and control (Oregon or *cp190*^3^/*tm*^*3*^) flies (Fig. 4A). A significant delay in pupariation was observed in flies bearing *cp190* LOF, confirming previous information on its role in 20E response.

Based on the role of CP190 in the 20E-dependent response in Kc cells, we selected genes whose transcription is dependent on both 20E and CP190, and analysed their transcription level in pupariated L3 larva and prepupa^29^. We observed a misregulation of selected genes in *cp190* LOF mutants (Fig. 4B). Transcriptional activation of *cg3394, e*23, *eip75b* and *bol* were found to be dependent on CP190. We observed a regulatory role of CP190 on *hph* through transcriptional repression. Altogether, these data further demonstrate that CP190 have a versatile input into regulation of 20E-dependent transcription. Considering its well-described role of an architectural protein, we presume that CP190 participates in the formation of a 3D structure of 20E-dependent loci complementing other activators and repressors.

## Discussion

The current study aimed to identify unknown molecular participants of the ecdysone-dependent transcription activation. Molecular mechanisms of transcription induction by 20-hydroxyecdysone have long been investigated^37^. To date many transcriptional regulators mediating ecdysone-dependent response have been described^14,38^. We conducted proximity-dependent biotin labelling experiments to find less prominent, but with potential functional significance. Two different systems were used: BioID2 long-term labelling and APEX2 rapid labelling^3^. Both labelling enzymes were linked to the EcR or Usp subunits which are the main mediators of the 20E response. Few previously described molecular participants of ecdysone-dependent transcription were found among the proteins precipitated by streptavidin resin. Both BioID2 and APEX2 precipitates contained several subunits of a nuclear pore (Nup358, Nup214, Nup88, Nup50, and Nup205), demonstrating its proximate location to EcR/Usp DNA-bound sites in the nucleus. Nuclear pore subunits were found to be involved in an enhancer-promoter interaction and nuclear memory of 20E-dependent genes^28^. Moreover, the Nup98 subunit was shown to co-precipitate with EcR^28^. Our data corroborate previous results, revealing an approximate location of the EcR/Usp heterodimer to a nuclear pore independently of transcriptional status (i.e., 20E treatment did not alter the amount of nuclear pore subunits in precipitations).

Nuclear pore subunits were shown recently to play an important role in chromatin decondensation, specifically, artificial tethering of Nups to chromatin sites causes SWI/SNF complex recruitment and nucleosome remodelling^39^. The results of our proximity-dependent biotin labelling experiments support a cooperative mechanism for nuclear pore and remodelling enzymes in ecdysone signalling. In addition to nuclear pore subunits, EcR/Usp-dependent biotin labelling resulted in precipitation of several chromatin remodelling complex subunits (Mor, Osa, Brm, Mi-2, and Kismet). All detected remodelling subunits were previously found to be involved in the ecdysone-dependent response. The most well-described participant of the 20E response, Mi-2, was shown to directly interact with EcR while preventing over-activation of 20E-dependent genes^27^. Both SWI/SNF subunits and Kismet were found to be recruited to promoters of 20E-dependent genes during their transcription after 20E treatment in *Drosophila* cells^15^. In addition, the SWI/SNF complex was shown to play a more complex role in the ecdysone cascade by participating in the 20E-dependent transcriptional repression of *ftz-f1*^40^. Our presented results fully support previous data of a functional involvement of SWI/SNF complex, NURD complex, and Kismet in the 20E-dependent response. A proximate location of these chromatin remodelling enzymes to the EcR/Usp heterodimer gives further evidence of a role of chromatin remodelling in 20E-dependent transcriptional regulation. This finding enhances understanding of molecular mechanisms of transcriptional regulation during development. Nuclear receptors of an ecdysone cascade were shown to play a role in chromatin remodelling at enhancers of developmental genes (i.e., maintain an opened or closed chromatin state)^41^. Only enhancers bound with ecdysone cascade proteins alter their chromatin state upon developmental transitions. Based on data obtained by Uyehara and colleagues, ecdysone signalling appears to be the primary driver of organismal development over time by promoting opening or closing of developmental enhancers through complex remodelling. Our current data indicate SWI/SNF, NURD, and Kismet remodelers as the main implementors those action leads to an effect of ecdysone signalling on chromatin in the regulatory regions of developmental genes.

A functional influence of nuclear architectural proteins on the 20E-dependent response has previously been demonstrated. Thus, 20E treatment of *Drosophila* cells was found to stimulate interaction between insulator and nuclear pore proteins, changing nuclear architecture^28^. It is often a challenge to describe the molecular mechanism for architectural proteins. Many insulator proteins do not colocalize with regulatory regions of genes and yet still affect their transcriptional output, likely through altering 3D DNA structure. Proximity-dependent techniques can be applied successfully in these cases. Our experiments on EcR/Usp-dependent biotin labelling revealed that the architectural proteins CP190 and Chromator are participants of a 20E-dependent response.

CP190 is a well-described cofactor of various *Drosophila* insulators, where its binding results in the functional output of an insulator protein complex^42–45^. CP190 was shown to be involved in 20E-dependent transcriptional regulation, mediating transcription of some 20E-dependent genes in *Drosophila* Kc cells^29^. The molecular mechanism of CP190 participation in 20E-dependent gene transcription was proposed to be through the looping of distant regulatory regions to the target promoter^29^. A direct connection between CP190 and a protein complex formed by EcR/Usp has not been suggested previously. The detection of CP190 in our proximity-dependent experiment corroborates previous information of its participation in the 20E response. We explored the relationship between the EcR/Usp heterodimer and CP190 insulator protein to understand its molecular mechanism using new techniques. Both BioID2- and APEX2-based proximity-dependent labelling indicate the proximate location of CP190 to EcR/Usp, identifying it as a stable associate of ecdysone-dependent loci. Its close proximity to EcR/Usp is independent of 20E treatment and persists long term, indicated by successful BioID2 labelling. In co-immunoprecipitation experiments, we found CP190 to be a part of a common protein complex formed by EcR with this interaction to be independent of the presence of 20E. CP190 functional importance in 20E-dependent gene transcription was demonstrated previously using a *Drosophila* Kc system^29^. In our work we confirmed these *in vitro* results *in vivo* in developing flies. We demonstrated that *cp190* LOF caused significant delay in puparium formation, a phenotype intrinsic to other EcR/Usp co-regulators^26^. We also detected a malfunctioning of 20E-dependent transcriptional response in *Drosophila* S2 cells and pupariated *cp190* mutant flies.

ChIP-Seq experiments showed the presence of CP190 at only a portion of EcR-bound sites and not at 20E-induced enhancers in *Drosophila* S2 cells. However, 20E regulatory regions (promoters and enhancers) were found to interact with CP190 sites via a looping mechanism. Hi-C data revealed that the regulatory hub, which is formed at 20E-dependent loci, consists not only of 20E-dependent promoters and enhancers but also of CP190-bound sites (possibly insulators), part of which is also bound with EcR. Short-term 20E-treatment (for 1 hour) did not change CP190 binding profiles in 20E-dependent loci according to our ChIP-Seq data. This is well correlated with a previous time-course analysis of CP190 binding in Kc cells (3-hour treatment with 20E), where it was shown that CP190 participates in stabilization of a basic regulatory hub at 20E-dependent genes in a hormone-independent manner^29^. The mode of CP190 action during 20E-dependent induction is likely to maintain a complex 3D structure at the loci enabling proper interactions between enhancers and promoters of genes, which appears to be transient and signal-dependent^31^. To illustrate all the possible ways of CP190 action at the 20E-dependent loci we provide a model of an average 20E-dependent gene (Fig. 4). Depending on the particular gene, CP190 can participate in regulatory hub formation out of (1) promoter, (2) common site bound both with EcR and CP190 or (3) distant CP190-bound site (by the looping mechanism).

The role of CP190 in regulation of 20E-dependent loci seems to be close to the previously described function of a nuclear pore complex in the ecdysone-dependent response^28^. The nuclear pore subunits were found to be stably associated with loci of 20E-dependent genes and promoting dynamic interaction between 20E-dependent regulatory regions inside the loci. It is interesting that nuclear pore subunits were found to constitute a common multiprotein complex together with EcR and CP190 independently of 20E-treatment thereby proving a functional link between nuclear pore complexes and CP190 in ecdysone signaling^28^.

The main finding of our study is that *in vivo* proximity-dependent labelling techniques can be a source of valuable information for researchers of transcriptional molecular mechanisms. This approach helped us to uncover several novel functional partners of EcR (Chro, Mor, Nup358, NELF A, and Spt5) as well as to additionally prove validity of previously identified partners (CP190 and Mi-2). We propose that *in vivo* proximity-dependent labelling can be a versatile screening tool to reveal unknown partners of regulatory proteins involved in transcriptional responses.

## Supplementary information

Supplementary information.

Supplementary information2
