# Supplementary material for: Proximity-dependent biotin labelling reveals CP190 as an EcR/Usp molecular partner
Source: Sci Rep. 2020 Mar 16;10:4793. doi: 10.1038/s41598-020-61514-0 (PMC7075897; doi:10.1038/s41598-020-61514-0)
Supplement: Supplementary file 1 — Supplementary information. [file 41598_2020_61514_MOESM1_ESM.pdf]

Proximity-dependent biotin labelling reveals CP190 as an EcR/Usp molecular partner

Marina Yu. Mazina[1], Rustam H. Ziganshin[2], Mikhail D. Magnitov[1,3], Anton K. Golovnin[1], Nadezhda E. Vorobyeva[1]*

[1]*Institute of Gene Biology, Russian Academy of Sciences, Moscow 119334, Russia*

[2] *Shemyakin-Ovchinnikov Institute of Bioorganic Chemistry, Russian Academy of Sciences, Moscow 117997, Russia*

[3] *Center for Precision Genome Editing and Genetic Technologies for Biomedicine, Moscow 119334, Russia*

** Corresponding author (email to vorobyeva@genebiology.ru)*

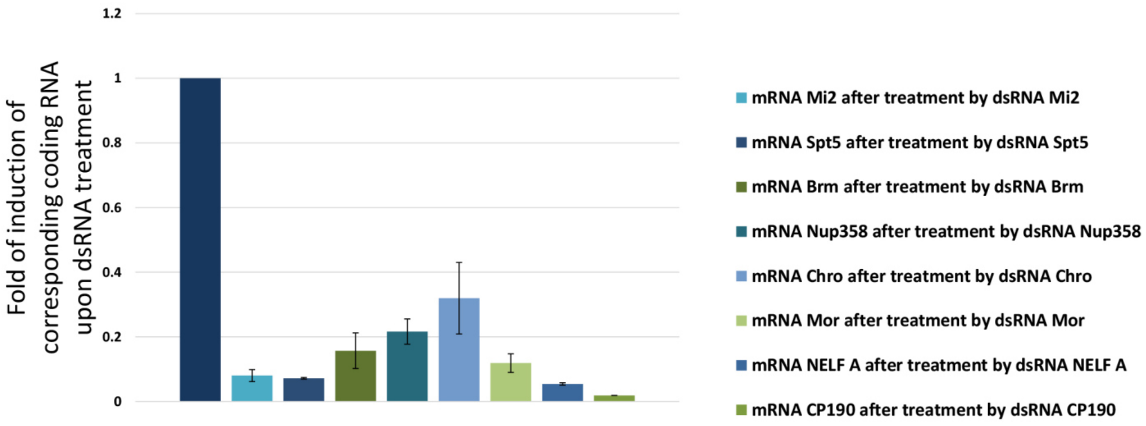

Fig S1. The test of efficiency of RNA interference-mediated knockdowns. Transcriptional levels were assessed by qRT-PCR. The Y-axis units represent fold of transcription induction. Data are mean values from three independent experiments, error bars represent standard deviations.

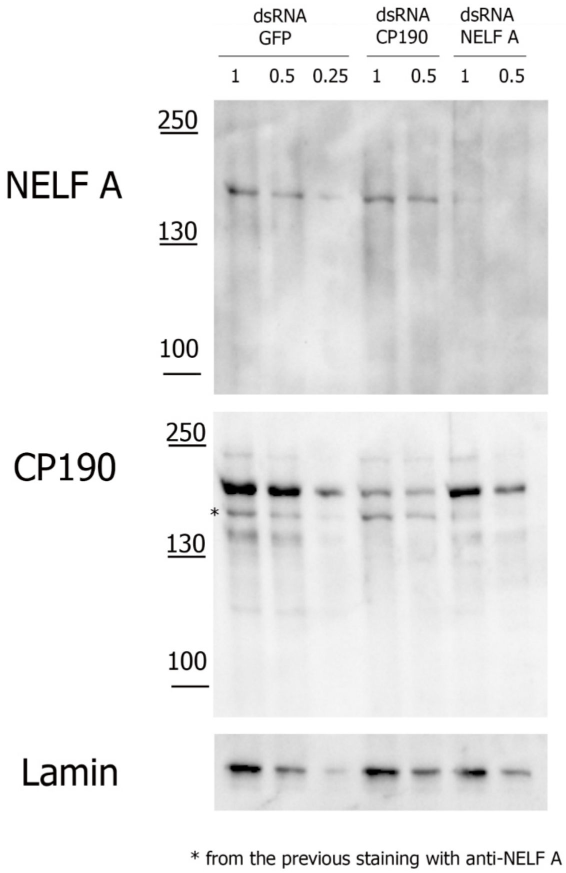

Fig S2. Western blots illustrating the specificity of anti-NELF A and anti CP190 antibodies. Nuclear protein extracts of S2 cells are loaded. CP190 and NELFA knockdowns were performed using corresponding dsRNA.
